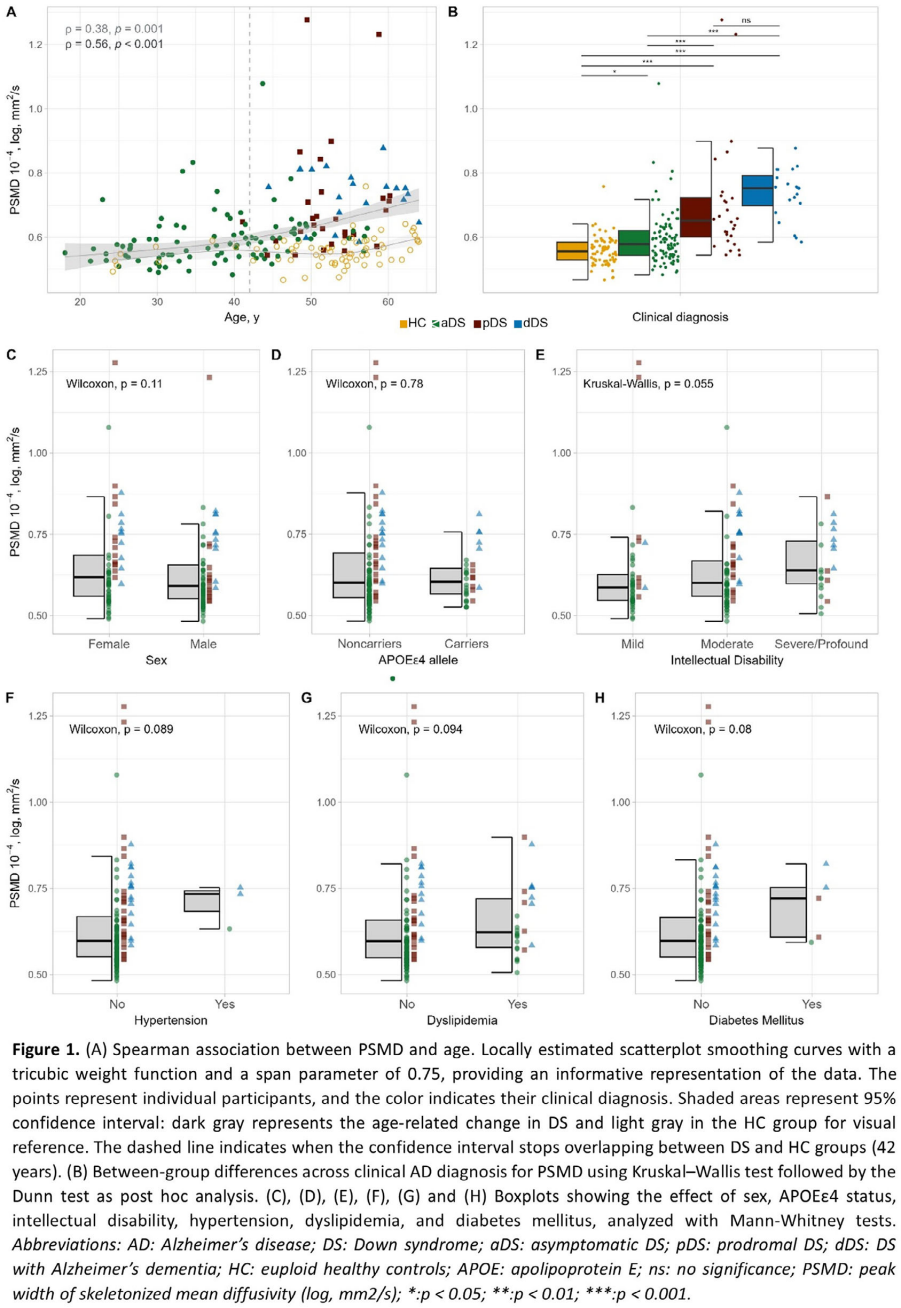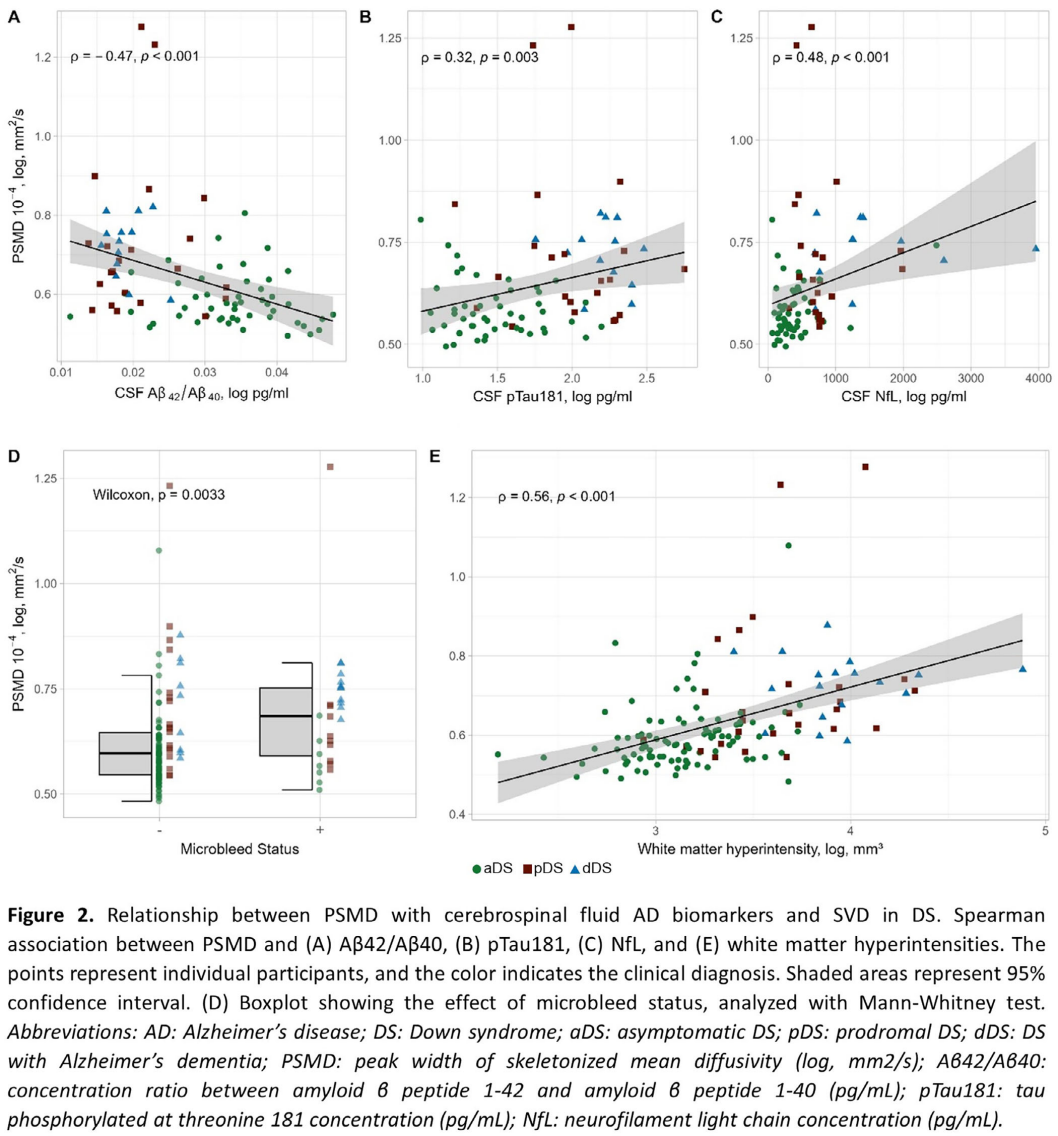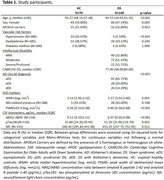# Early Detection of White Matter Integrity Changes in Down Syndrome: The Promise of PSMD as a Sensitive Biomarker

**DOI:** 10.1002/alz70856_106537

**Published:** 2026-01-08

**Authors:** Alejandra O. Morcillo‐Nieto, José Enrique Arriola‐Infante, Maria Franquesa‐Mullerat, Sara E Zsadanyi, Lídia Vaqué‐Alcázar, Mateus Rozalem Aranha, Jose Allende Parra, Zili Zhao, Javier Arranz, Íñigo Rodríguez‐Baz, Lucía Maure‐Blesa, Laura Videla, Isabel Barroeta, Laura Del Hoyo, Bessy Benejam, Susana Fernandez, Aida Sanjuan Hernandez, Sandra Giménez, Daniel Alcolea, Olivia Belbin, Alberto Lleó, Maria Carmona‐Iragui, Juan Fortea, Alexandre Bejanin

**Affiliations:** ^1^ Center of Biomedical Investigation Network for Neurodegenerative Diseases (CIBERNED), Madrid, Spain; ^2^ Sant Pau Memory Unit, Hospital de la Santa Creu i Sant Pau, Biomedical Research Institute Sant Pau, Universitat Autònoma de Barcelona, Barcelona, Spain; ^3^ Department of Medicine, Faculty of Medicine and Health Sciences, Institute of Neurosciences, University of Barcelona, Barcelona, Spain. Institut d’Investigacions Biomèdiques August Pi i Sunyer (IDIBAPS), Barcelona, Spain; ^4^ Neuroradiology Section, Department of Radiology, Hospital de la Santa Creu i Sant Pau, Biomedical Research Institute Sant Pau, Universitat Autònoma de Barcelona, Spain, Barcelona, Spain; ^5^ Barcelona Down Medical Center, Fundació Catalana Síndrome de Down, Barcelona, Spain; ^6^ Multidisciplinary Sleep Unit, Hospital de la Santa Creu i Sant Pau, Barcelona, Spain; ^7^ Sant Pau Memory Unit, Hospital de la Santa Creu i Sant Pau, Biomedical Research Institute Sant Pau, Barcelona, Spain

## Abstract

**Background:**

Down syndrome (DS) is a genetic condition associated with an ultra‐high risk of developing Alzheimer's disease (AD). While vascular risk factors (VRF) are less prevalent in DS, radiological hallmarks of small vessel disease (SVD) are commonly observed. The peak width of skeletonized mean diffusivity (PSMD) is a diffusion tensor imaging (DTI) marker designed to quantify the white matter (WM) damage secondary to cerebral SVD. Here, we aimed to characterize PSMD alterations along the AD continuum in DS and define associations with AD and SVD biomarkers.

**Method:**

Cross‐sectional study using the Sant Pau Initiative on Neurodegeneration (SPIN) and Down Alzheimer Barcelona Neuroimaging Initiative (DABNI) cohorts. We included 70 euploid healthy controls (HC; median age [IQR]=54.37 [9.03] years, females=70%), and 140 individuals with DS (age=44.15 [17.33] years, females=47.14%), including 92 asymptomatic (aDS), 28 prodromal AD (pDS), and 20 with AD dementia (dDS), underwent a 3T‐MRI protocol. DTI images were visually inspected, and PSMD was extracted using a fully automated pipeline (Baykara et al., 2016). Non‐parametric tests were used to assess the effect of sociodemographic (age, sex, intellectual disability) and genetic (APOE haplotype) factors, VRF (hypertension, dyslipidemia, diabetes mellitus), and AD disease severity on PSMD. Associations with AD biomarkers (Aβ42/Aβ40 ratio, pTau181, and NfL in cerebrospinal fluid [CSF]) and SVD markers (WM hyperintensities [WMH] and microbleeds) were evaluated.

**Result:**

PSMD was more strongly associated with age in DS (rho=0.56; *p* <0.001) than in HC (rho=0.38, *p* <0.001; Figure 1A), with regression lines starting to diverge at age 42. No significant effects were found for sex, APOEε4 status, intellectual disability, or VRF (Figure 1C‐D‐E). PSMD increased linearly along the AD continuum (HC<aDS<pDS=dDS; Figure 1B), and correlated with CSF biomarkers of AD and neurodegeneration (Figure 2A‐B‐C). PSMD increased significantly in DS individuals with at least one microbleed (*p* = 0.003, Figure 2D) and strongly correlated with WMH (rho=0.56, *p* <0.001; Figure 2E).

**Conclusion:**

In DS, PSMD changed approximately 10‐15 years before the onset of AD symptoms and linearly increased with AD clinical stages, AD pathology, and SVD metrics. These findings underscore the potential of PSMD as a sensitive biomarker for detecting early WM alterations in adults with DS.